# *“When the Fun Stops*, *Stop”*: An analysis of the provenance, framing and evidence of a ‘responsible gambling’ campaign

**DOI:** 10.1371/journal.pone.0255145

**Published:** 2021-08-26

**Authors:** May CI van Schalkwyk, Nason Maani, Martin McKee, Samantha Thomas, Cécile Knai, Mark Petticrew

**Affiliations:** 1 Faculty of Public Health and Policy, London School of Hygiene and Tropical Medicine, London, United Kingdom; 2 SPECTRUM Consortium, London, United Kingdom; 3 School of Public Health, Boston University, Boston, MA, United States of America; 4 Faculty of Health, Institute for Health Transformation, School of Health and Soc. Dev., Deakin University, Geelong, Australia; University of Toronto, CANADA

## Abstract

*When the Fun Stops*, *Stop*, is a prominent ‘responsible gambling’ campaign in the UK, originally funded and delivered by the industry-initiated and funded Senet Group. Since the Senet Group’s dissolution in 2020, the campaign has been overseen by the Betting and Gambling Council (BGC), the main gambling industry trade body. There has been no prior analysis of the activities, ideas and framing adopted by the Senet Group, who claimed to be acting as an industry ‘watchdog’ and oversaw what they characterised as a major public education campaign. We collated written and image-based material related to the Senet Group and its *When the Fun Stops*, *Stop* campaign from multiple sources. Guided by Entman’s four functions of framing, we analysed the Senet Group’s framing of the issues it sought to address, particularly harmful gambling, as well as its causes, and the solutions, focusing on the group’s main activity: the delivery of the *When the Fun Stops*, *Stop* campaign. We also critically appraised an evaluation of the campaign funded by the Senet Group, using the findings to interrogate the stated claims about the campaign’s effectiveness. The analysis showed that the Senet Group’s framing of the problem, its causes, and proposed responses resemble those adopted by other industries and industry-funded groups. This involves portraying any harms caused by their products as limited to an atypical minority, rejecting upstream determinants of harm, and promoting individually-targeted voluntary measures, all contrary to the evidence of what works in health promotion, and what would characterise a public health approach. Neither the existing evidence base nor the evidence presented by the Senet Group support their claims about the campaign’s effectiveness. These findings add to concerns about industry-funded campaigns in other areas. To minimise conflicts of interest, interventions intended to address gambling-related harms, such as public education campaigns, should be evidence-based and developed, implemented and evaluated completely independent of the industry and industry-funded organisations.

## Introduction

The global liberalisation of commercial gambling over the past forty years has been justified by governments and industries as increasing choice for consumers while creating jobs and government revenue [1, 2]. However, it has also been associated with considerable harms to individuals, families and communities [3]. Despite promises of a shift in the regulatory environment in the United Kingdom (UK) with the Gambling Act 2005 currently under review by the Conservative government [4], policy remains dominated by the concept of ‘responsible gambling’ [5], as in many other high-income countries.

The philosophy underpinning responsible gambling has been challenged in the academic literature [6–9]. Concerns have been raised about how it enables a shifting of responsibility onto the individual to gamble safely, helped by limit-setting, self-exclusion, and industry-funded awareness/education campaigns, while deflecting attention from the efforts of the industry to recruit and retain gamblers, the limits of industry self-regulation, the impacts of liberalising policies, and the risks posed to democratic policy-making by the establishment of close relationships between governments and the gambling industry [1, 6–8, 10–12]. Instead there are those who support the adoption of a public health approach [2, 9, 13], which recognises the role played by gambling policies, environments and industry practices in contributing to gambling harms, and that individual measures are often ineffective [14] and stigmatising, thereby contributing to harmful stereotypes of people who experience gambling problems [15, 16].

A public health approach is also informed by a growing understanding of the commercial determinants of health. This perspective is informed by a body of literature documenting the strategies adopted by different industries selling potentially harmful products to delay regulation by spreading doubt and placing responsibility for harm onto individuals, including the funding of industry-friendly research and public education campaigns, with the latter predominantly focused on individualised determinants of, and solutions for, the problems associated with their products, including gambling [12, 17–21]. This body of research also details the implications for public health posed by corporate political strategy, including expanding understanding of the consequences of the conflicts of interest that can arise as a result of corporate involvement in policy development, research, and the delivery of interventions and information [10, 17, 20, 22, 23]. These considerations, coupled with growing recognition of the broad socio-cultural, environmental, commercial and political factors leading to normalisation of gambling, and subsequent gambling related harms [2, 24] have led to calls for the establishment of a public health response to gambling harms in the UK [25, 26]. However, to date, most interventions to tackle gambling harm in the UK have been led by the industry or industry-funded bodies and focus on problematising and changing individual behaviour. Independent research on campaigns funded by manufacturers of other harmful products, such as alcohol and tobacco, has repeatedly found these types of campaigns to be ineffective, misleading, or promoting the company or its products [27–30]. For example, a recent Australian study compared alcohol harm reduction advertisements developed by public health agencies and alcohol industry Social Aspects/Public Relations Organisations (SAPROs), finding that the latter were less effective at stimulating motivation and intention to reduce consumption of alcohol, and incited more positive fun-related perceptions towards those who drink alcohol [31].

### The Senet Group and the *When the Fun Stops*, *Stop* responsible gambling campaign

In 2014 four of the UK’s then largest gambling companies (William Hill, Ladbrokes, Coral, and Paddy Power) formed the Senet Group “… in response to public concerns on gambling, and gambling advertising in particular” [32]. Its website described the group as:

“… an independent body set up to raise standards in the sector, supporting the Gambling Commission’s work to make services safer and fairer ensuring, in particular, that responsible gambling messages are put to players with frequency and prominence” [32].

**Fig 1:**
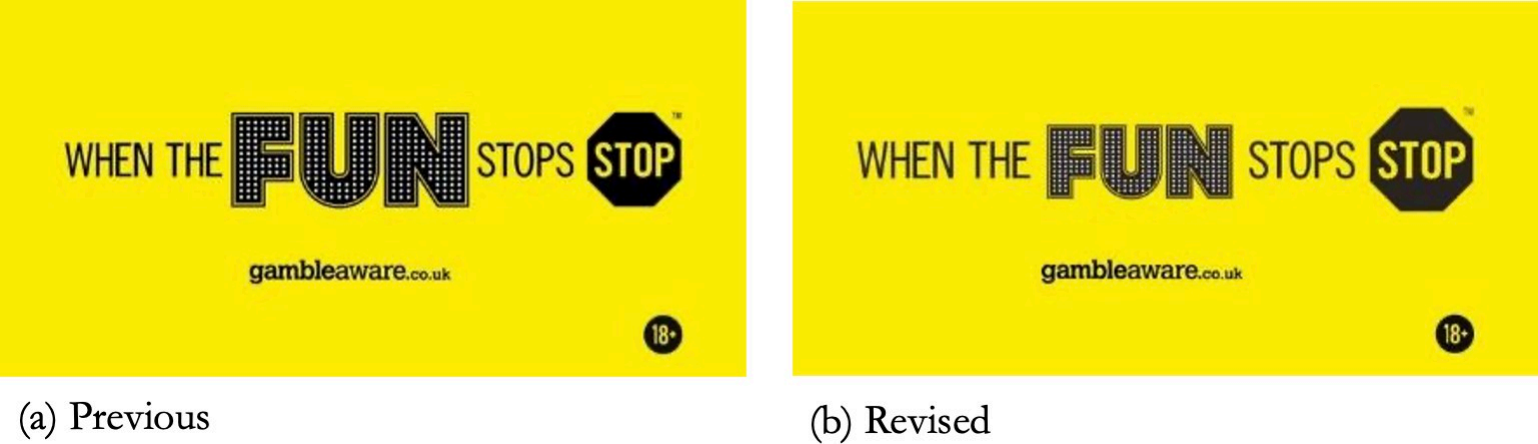
The Senet Group’s responsible gambling campaign tagline. In the original campaign imagery (a) the font size for the word “FUN” was larger and the second “STOP” was smaller than the version currently in use. On the 1^st^ of June 2015, the Senet Group announced that “Senet has slightly altered the relative size of the words ‘Fun’ and ‘Stop’ in its yellow advertising strip, ‘When the Fun Stops, Stop’, to bring the two symbols into better balance” (b) (Source: Senet Group press release titled *Senet Group runs new burst of #BadBetty advertising and strengthens regulation*) [73].

Its activities were overseen by a Board which had responsibility for its operation, comprised of “two members from the gambling industry, two lay members from outside the industry and an independent Chair, who will act as Standards Commissioner” [33]. Its main public education activity was a responsible gambling campaign promoting the tagline *When the Fun Stops*, *Stop* (Fig 1) [34]. Initiated in 2015, this campaign has three main elements, the tagline itself, responsible gambling “tips”, and “Bad Betty” advertisements [35–37]. The campaign was created by The Corner, a London-based advertising agency whose clients include gambling and sugar-sweetened and alcoholic beverage companies, among others [36]. The Senet Group described the campaign tagline as serving “…to highlight the warning signs of problem gambling and the benefits of staying in control” [37]. The tips are provided “…to help prevent gambling becoming a problem” [37]. Campaign imagery is displayed in shop windows and on static, televised, and online gambling advertisements, including on social media. According to the Senet Group, in 2018 its messaging was displayed by approximately 40% of UK gambling companies [38]. A prevalence of approximately 40% was similarly reported by a content analysis conducted in 2018 on paid-for gambling advertising featuring on eight UK media channels [39]. It also features on sleeves of football players’ jerseys, introduced as part of “a new responsible gambling campaign” proposed by Sky Bet and the English Football League [40].

In April 2020 the Senet Group was dissolved and all of its assets and responsibilities, including the campaign, were transferred to the Betting and Gaming Council (BGC), the newly formed industry standards body launched in 2019 [41]. The campaign continues to run in the UK, and has featured in safer gambling messaging shown throughout the COVID-19 pandemic. Increased dissemination of safer gambling messaging formed part of the ten pledges made by the BGC and its member companies to keep players safe during the pandemic when players are potentially at greater risk of harm due to stress, isolation, financial difficulties, and increased use of personal electronic devices such as laptops and smartphones. Upon launching the pledges on 27^th^ March 2020 the BGC stated that:

“Although overall gambling has fallen dramatically with the absence of sport and due to the closure of betting shop and casino closures, the BGC’s pledges will come into force immediately to help ensure that the highest safeguards are in place and action is taken to protect anyone betting online who may be more vulnerable as a result of the crisis” [42].

The Senet Group had previously commissioned an evaluation of the campaign based on a repeat cross-sectional online survey of adults (18+), undertaken by Bilendi, a market research company, and performed bi-annually over the period 2015–2017 (sample sizes ranging from 2000 to 2015 adults) [43]. Based on this evaluation, the Senet Group made several claims over multiple years and in different fora about the effectiveness of the campaign. In 2018, the Advertising Standards Authority (ASA) rejected the Group’s claim, made in *The Week* magazine, that the campaign was effective in reducing harm [44]. One pre-print study suggests that, based on an incentivised survey of people who identify as football fans and have experience with online sports betting, the campaign has had little or no effect on the amount they bet [45]. A second pre-print study, using larger samples of participants, higher stakes and more realistic tasks, again demonstrated evidence of either no beneficial effect or an increase in the proportion of money bet when participants were exposed to the yellow-coloured version of the campaign’s messaging compared to a no-message control [46]. Furthermore, Gambling Commission CEO, Neil McArthur, questioned the effectiveness of the campaign, as well as the independence of its evaluation; “…reading claims about the effectiveness of the campaign by the same marketing team that invented it doesn’t carry much weight in my view” [47]. Despite these concerns, industry-funded mass media campaigns continue to be a cornerstone of the UK’s approach to addressing gambling harms, evidenced by the launch of another campaign, *Bet Regret*, developed by the industry-funded charity GambleAware [48], which has been criticised for its methods and industry funding [26, 49].

In light of these considerations, important questions remain: what were the ideas, problem definitions, and causal mechanisms adopted by the Senet Group that informed its work, including the design and content of the campaign as an intervention to address the problem? What evidence was drawn upon and what justifications were used to assert its effectiveness and continued dissemination? Drawing on a theoretical framework informed by framing theory, corporate strategy, and commercial influences on health, we therefore analyse the framing adopted by the Senet Group to (1) conceptualise and define the problem(s) and its causes, (2) propose solutions deemed acceptable and effective, and (3) describe the nature and effectiveness of the campaign. We also critically appraise the Senet Group-funded evaluation of the campaign and, based on our analyses, seek to determine whether the Senet Group’s framing and the campaign is consistent with a public health response to gambling harms.

### Methods

The analysis involved two stages: (1) a framing analysis of campaign materials (“the data”), and (2) a critical appraisal of the Senet Group-funded campaign evaluation.

### Data

One researcher (MvS) identified and collated material related to the Senet Group and the campaign from multiple sources [23]. Written and pictorial content from the Senet Group and campaign-specific websites (material was extracted before these websites were shut down in 2020; where available we have referenced web archives), advertisements, and radio and YouTube interviews were obtained prior to the Senet Group’s dissolution, and transcribed where necessary. Campaign evaluation reports were downloaded from the Group’s website. Factiva [50], a global news database, was used to identify relevant news articles using the search term “when the fun stops, stop” and published up to 29^th^ June 2019 (no limits applied). Additional material, for example, Twitter content, consultation responses, and annual reports, was identified using snowballing and interrogation of references.

### Framing theory

The framing analysis was informed by framing theory and methods used in related fields, including tobacco, obesity and alcohol [51–53]. Framing is powerful, serving to shape how an issue is defined and, as a consequence, the policies and interventions proposed and supported [52, 54]. As described by Entman, framing brings some aspects of an issue to salience, while silencing others, and serves four functions; “…to promote a particular problem definition, causal interpretation, moral evaluation, and/or treatment recommendation” [55]. A framing analysis involves the interrogation of the framing adopted by different actors, the values and ideas that underlie their framing, and how framing is employed within the context of a contested policy issue to further the interests of particular individuals or parties [54].

### Analysis

We analysed how the board of the Senet Group and its industry backers (herein referred to as the Senet Group) employed framing to define the problem(s), provide causal explanations, assign moral judgements, and prescribe suitable interventions. Understanding how the problem is framed and conceptualised by those claiming to act as an industry “watchdog” and overseeing the campaign is important as this is often designed to legitimise the chosen solution, in this case, the campaign, and influence its uptake and acceptance by the public and policymakers. Framing relating to gambling, including but not limited to the campaign, was analysed using Entman’s four framing functions [55]. The data were analysed using conceptual coding and abductive reasoning, working iteratively between findings and framing theory [52, 54]. This allowed for the identification, and provided deeper understanding, of the framing adopted by the Senet Group. An inductive approach was applied, given the paucity of previous research focused on gambling industry-funded organisations. The analysis was conducted independently by two researchers (MvS and MP), who systematically read and open-coded all material. Coding and emergent framings were discussed to reach consensus among the researchers and discussion with a third researcher (NM) resolved any disagreement. The content of campaign-based static images and film were similarly analysed, using the approach described by Bohnsack [56], whereby images were treated as distinct from text, with explicit and implicit knowledge portrayed by the imagery integrated into the overall context, thereby complementing findings from the text.

### Critical appraisal of evidence from the Senet Group-funded evaluation of the campaign

We identified five evaluation documents available at the time from the Senet Group Website (now defunct). The evaluation documents, prepared by Bilendi for the Senet Group, report on the effects of the campaign on awareness, recognition of campaign phrases, and self-reported prompts to behaviour change (i.e. gamble more responsibly). Using a standard critical appraisal tool for the assessment of cross-sectional studies [57], two researchers (MvS and MP) assessed the robustness of the survey methods, results, and conclusions drawn, as well as procedural aspects including research governance and peer review.

Ethical approval was not required as the research involved secondary analysis of publicly available data.

## Results

Table 1 summarises the volume and type of material identified and analysed.

**Table 1:** Summary of documentary materials collated and included in the analysis.

Material type	Description	Source
Website content	Online content from the Senet Group and campaign websites	https://senetgroup.org.uk (now available via Internet Archive WayBack Machine https://web.archive.org/web/20150520154617/http://senetgroup.org.uk/) http://www.whenthefunstops.co.uk (No longer functioning)
Radio interviews	Five radio interviews with Senet Group Chair or Chief Executive	https://senetgroup.org.uk (now available via Internet Archive WayBack Machine https://web.archive.org/web/20150520154617/http://senetgroup.org.uk/) https://www.bbc.co.uk/radio/play/b0bhfj0y
Campaign ads	Two ads accessed through campaign website and YouTube: “Bad Betty Football” & “Bad Betty Betting Shop”	http://www.whenthefunstops.co.uk (No longer functioning) (films still available here https://www.thecornerlondon.com/work/senet-when-the-fun-stops-stop/)
Promotional films	Campaign-related promotional films accessed via YouTube: One interview based, “Gambling industry leaders back Senet Group’s When the Fun Stops, Stop campaign” and one delivered by Sky Bet “Sky Bet–When the fun stops, stop”	https://www.youtube.com/watch?v=5ZlRn87p38Q
		https://www.youtube.com/watch?v=mblDBu8i5Gc
GIF files	11 unique GIF files accessed through the Senet Group twitter account	@SenetGroup (now via Internet Archive WayBack Machine https://web.archive.org/web/20170512092843if_/https://twitter.com/senetgroup)
PDF and Word documents	1 x annual report, 1 x consultation cover letter, 3 x consultation responses, 5 x evaluation reports, 1 x advertisement	https://senetgroup.org.uk (now available via Internet Archive WayBack Machine https://web.archive.org/web/20150520154617/http://senetgroup.org.uk/)
Mass media articles	Factiva, a global news database, search: 181 results retrieved	https://professional.dowjones.com/factiva/

### Framing analysis

Emergent framings are presented in sequence from the broader framing adopted by the Senet Group to focusing on those specific to the campaign and claims about its impact: (1) *Framing the problem*: framing adopted by the Senet Group to portray gambling, gambling regulation, gamblers and to define gambling harms, provide causal explanations for these harms, make moral judgements, and prescribe solutions, (2) *The Campaign*: framing of the campaign’s aims, and theories or evidence used to inform its design and (3) *Evidence and impact*: claims of effectiveness and causation.

### Framing the problem

#### “Millions” versus the minority

When conceptualising the issue of gambling harms, the Senet Group contrasted the large majority of gamblers who did so safely, for fun and/or leisure, with a small minority of vulnerable gamblers who lack control and understanding, and are harmed by gambling. For example, in 2015, its Chair stated on BBC Radio Sheffield:

“… the reality is that a very small number of people get into a great deal of trouble and what I think is necessary is that the gambling industry takes that seriously, … says what can we do to help, and what the Senet Group tries to do, is to do that by just giving people warnings, giving them messages so that they understand” [58].

This portrays gambling as providing entertainment to a large number of people, with individuals responsible for stopping when they are no longer having fun. According to this framing, gambling is a freedom to be enjoyed responsibly, regularly, and safely, by a large collective. For example, in a joint letter published in national and regional newspapers, the CEOs of the Senet Group’s founding companies said “Every day, millions of us place a bet–a freedom that should be enjoyed safely and responsibly” [59].

#### Us and them

Gamblers experiencing harm are framed as problematic users with individual vulnerabilities:

“The Senet Group argues that the focus, in terms of outcomes, should be on any impacts broadcast advertising might have in relation to those who are vulnerable in terms of moving from social to problem gambling,…” (Senet Group response to the 2016 Call for Evidence: Review of Gaming Machines and Social Responsibility Measures, Department of Digital, Culture, Media and Sport, UK Government) [60].

This presents them as extreme and exceptional cases, as seen in stories about individuals who have stolen money from family members or who have died by suicide, such as:

“We did some research among gamblers and you know most people enjoy a little flutter or you know a bit of a gamble, you know, most of the time but there are some, like Anne’s son, who, you know, can slip down a path towards addiction and what our research showed was that those people found that gambling wasn’t fun anymore they weren’t enjoying it and that was an early signal. …” (Senet Group Chief Executive, BBC Radio Sheffield) [61].

In these ways, people who experience gambling addiction are presented as differing from the majority of people who gamble, creating a conceptual contrast between “us” (the majority who gamble responsibly and within our limits), and a vulnerable and, by implication, weaker minority of people who have lost control, lack willpower, or have an inherent susceptibility to addiction. This has the effect of framing gambling harm—the result of an encounter between a normal product and flawed individuals–as lying outside of industry control. For example, in a BBC Radio Sheffield interview the Senet Group Chair stated:

“I wanted to talk to people who had lost hundreds and thousands of pounds, who’d lost their homes and so on. I wanted to understand whether there was something that the industry was doing that made that happen and my conclusion was that it wasn’t, that there are some people who are much more vulnerable to gambling, to drinking and so on and funnily enough they sometimes go together, and that they have issues that need to be addressed professionally” [58].

#### Mechanisms and solutions

Framing the problem as one of individuals leads to certain “solutions”; by framing the problem as ‘problem individuals’, the solutions are inevitably individually-focussed, avoiding anything that confronts problems with the industry or its products. For example, these framings often invoke the need to help people stay “in control”, with education seen as key to helping them do so. In some examples this involved simply helping people to identify their risky gambling behaviours and to restore or maintain control:

“Absolutely, I think Graham is a model to anyone who is thinking of gambling, … he has a friendship with gambling, he’s not addicted to it, he could give it up at any time, he is using self-discipline, keeping it in control, not betting more than he can afford, not spending more time than he can afford and getting a lot of pleasure out of it.” (Senet Group Chief Executive, BBC Radio Scotland) [62].

The “in control” theme also provided the title for one of Senet Group’s research reports, *In Control*: *How to support safer gambling using a behaviour change approach* [63], about which their Chair said:

“This research report provides some practical insights into … how the gambling industry might support their enjoyment of gambling by helping them stay in control” [64].

This “in control” framing supports solutions based on promoting personal responsibility to gamble in “moderation”. In response to consultation on proposals for changes to gaming machines and social responsibility measures 2017/18, Department of Digital, Culture, Media and Sport (DCMS), UK Government, the Senet Group claimed the sector should own, and take responsibility for, “…encouraging moderation and good sense when customers use their services…” [65].

This logic builds upon the ‘us and them’ theme, translating into a ‘fun/not fun’ dichotomy: that gambling is fun for “us”, the majority, who are “in control”, but gambling can switch to not being fun for those who lack control or self-discipline, and as a result of this weakness or vulnerability can “slip” into addiction.

#### Industry self-regulation

The Senet Group’s proposed solutions to gambling harm promoted industry involvement and ownership, and rejected statutory regulation. Self-regulation is portrayed as efficient and impactful, supporting restoration of individual good sense, moderation, and responsibility. In a cover letter accompanying their response to the 2016 Call for Evidence: Review of Gaming Machines and Social Responsibility Measures (DCMS, UK Government), voluntary measures were framed positively, using concepts such as quality, speed of delivery, and low cost to consumers and others:

“We see a need for the Government, Gambling Commission and other stakeholders to reflect on the mechanics for “delivering” actions which reflect social responsibility. Some cannot be set or secured through statutes and regulations. Increasingly, they rely on cultural and behavioural change, rather the [sic] specific regulatory actions. Often this will be through individual or collective voluntary action, through forms of self-regulation on the part of operators and agreements or partnerships with advertisers, broadcasters and others. The Commitments made in 2015 by Senet Group members and complied with consistently are proof that non-statutory solutions that build on and build-in industry commitment can deliver better, faster and at less cost to the taxpayer and consumer” [66].

Their approach to regulation draws heavily on this ‘industry-as-part-of-the-solution frame’, with Senet Group members stressing the need for industry involvement to achieve optimal outcomes: “The best results are achieved when those who are regulated help inform the shape of that regulation.” (Senet Group Chief Executive in response to release of new gambling advertisement regulations) [67].

### The campaign: *When the Fun Stops*, *Stop*

#### Ambiguity, mixed messages and shifting aims in the campaign

The Senet Group’s framing of the campaign sought to portray gambling as normally good (an enjoyable experience), while accepting there can be a risk, as in “…an advertising campaign reminding gamblers that when gambling stops being fun then, they should call it a day” [68], while at other times their core message was described as “…that betting more than you can afford, or betting when you’re getting angry or frustrated, is a ‘Bad Betty’ and you should think again” [69] or “…to build understanding of the risks if gambling moves from being a social activity into something serious” [70].

Previous industry-funded responsibility campaigns, for example by the alcohol industry, have used vague and ambiguous wording, potentially sending mixed-messages by both apparently promoting the product while simultaneously appearing to warn about consumption [28, 29, 71]. This campaign also employs seemingly ambiguous wording and phrases, exemplified by the campaign tagline; *When the Fun Stops*, *Stop*. As highlighted above, the tagline is framed as conveying multiple different messages: “…..pause and think about his actions” [72], “…they should call it a day” [68], and “…When the Fun Stops Stop, and what it shows was that young guys are betting and in situations where they need just to stop and calm down and that’s sorts of messages I think will help people” [58].

#### Fun, humorous and joking tone

Elements of the campaign (e.g. music, imagery, and written content) adopt a humorous or light-hearted tone that can also convey mixed messages. For example, the GIF “never chase your losses”, tweeted by the Senet Group, contained a moving image of a white-haired fluffy puppy chasing its own tail. When presented as a moving image, the word “FUN” from the campaign tagline appears before the remaining words at various times and is accompanied, and emphasised, by twinkles or flashes. In June 2015, it was announced that “Senet has slightly altered the relative size of the words ‘Fun’ and ‘Stop’ in its yellow advertising strip, ‘When the Fun Stops, Stop’, to bring the two symbols into better balance” [73]. However, the word “FUN” is still in a larger font than the word “STOP”. Also, the tagline’s stop sign is not a typical UK stop sign as would perhaps be widely recognised, being black and hexagonal, as opposed to the standard UK stop sign (circular and red, with a diagonal slash).

In a 2018 BBC Radio 5 live interview, the Senet Group Chair did acknowledge that concerns about the approach adopted by the campaign had been raised, but then countered them by arguing that a different approach is likely to backfire: “It’s a challenge, I mean some people say for example we shouldn’t have the word “When the Fun Stops, Stop” we shouldn’t have the word fun, but if you lecture people you can have the opposite effect” [74].

### Evidence and impact

#### Health messaging: Claims of effectiveness

The Senet Group framed the campaign, its content, and approach, as being consistent with methods of health messaging and awareness-raising known to be effective. In particular, the campaign and surrounding discourse emphasised the effectiveness of humorous content used repeatedly across multiple platforms:

“We have found the best results are achieved when the messages are written in informal language and communicated consistently across an assortment of platforms. We do not preach or seek to scare, but rather speak in a way that will encourage gamblers to consider their own habits and to speak to family members and friends if they think they might need help.” (Senet Group Chair upon release of the March 2017 campaign evaluation report) [70].

Their claims did, however, reveal apparent confusion about who they were targeting: “We are clear, however, that the light and conversational tone which has worked with the generality of players with gambling problems is not appropriate in messaging activity that is targeted specifically at problem gamblers.” (Senet Group response to the DCMS 2017/18 consultation) [65] Yet “Senet’s approach to consumer engagement reflects the lessons learnt by public information campaigns over the past 20 years, that positive messaging is more likely to resonate with our target cohort of young men between the ages of 18 and 24, where research indicates that they are more likely to be problem gamblers.” (Senet Group website) [38].

#### Campaign effectiveness: Claims of causal effects

The effectiveness of the campaign was often framed in terms of its impacts on awareness and education, assuming causal associations between the campaign and behaviour change. For example, the Senet Group claimed that “…the campaign has helped over a third of regular gamblers control their gambling, and more than two million people have directly quoted ‘when the fun stops, stop’” [72]. This claim of success recurs frequently, as in when the Senet Group makes reference to “…the positive impact this [the campaign] has had on many” [66] and that “Our ’Bad Betty’ adverts continue to be very successful in educating gamblers to become more aware of the signs behind problem gambling, as well as encouraging a more responsible approach” [72]. Their written submission to the Government’s 2017/18 consultation states that

“Today, surveys show over 80% of players are aware of the messages and tips in the Senet Group’s responsible gambling campaign and it seems clear these have resulted in millions of players changing their behaviours and millions of others feeling more confident to raise gambling issues with a friend or family member” [65].

Frequent claims were also made about effects on awareness and self-reported behaviour, including “…to approach gambling more responsibly” [70].

The success of their approach is presented, at least implicitly, as self-evident, as in the promotional film for the campaign, where the Senet Group Chair states that “…when the fun stops, stop works because it’s simple, people understand it, they like it and its effective and it changes behaviour” [75]. This assumes that campaign exposure must have an effect despite a lack of objective measures of behaviour change. They even portrayed the campaign and its impact as beyond expectations or even superior to other campaigns: “The reach and impact of this campaign is at levels few if any other public health awareness and behaviour change can match” [43].

### Absences

As emphasised by Entman [55], what is absent within frames is as important as what is present. Notably, despite the campaign being framed as serving “to build understanding of the risks if gambling moves from being a social activity into something serious” [70], we could find no evidence that the campaign addresses certain known risks associated with harmful gambling, such as depression, suicide, homelessness or domestic violence, or distinguishes between the risks of different gambling products [2].

### Critical appraisal of evidence from the Senet Group-funded evaluation of the campaign

We located five campaign evaluation documents from the Senet Group website, covering the period 2015–2017 (an evaluation report for the latter half of 2015 could not be located). Using the AXIS tool for critically appraising the quality of cross-sectional studies [57], we critically appraised the evaluation, although this was difficult given the reports’ brevity and sparse content relating to methods, analysis or findings (S1 File). This identified several weaknesses in study design and conduct, and a risk of bias and conflicts of interest. It also challenged the extrapolation of the findings to the adult population as a whole. Although the samples are referred to as nationally representative, no weighting appears to have been performed and it is not clear what is meant by “representative”, as in representative of the adult population or adult gambling population, for example. We found no evidence to substantiate the claims made by the Senet Group about the effectiveness of the campaign in delivering behaviour change, either for existing problem gamblers or those at risk of becoming so, or in comparison to other comparable public health campaigns.

## Discussion

Our analysis demonstrated that the Senet Group framed gambling as a leisure activity which is undertaken safely by the majority of gamblers. Gambling was dichotomised: fun (safe) or not fun (unsafe). Those that are harmed, described as a minority, have “lost control”, no longer have “fun”, and are presented as vulnerable in some shared way which is independent of their engagement with gambling. Prescribed solutions were based on industry-led provision of information on responsible use. This framing aligned with the design of their public awareness campaign. The simplistic dichotomy presented by the Senet Group—fun (safe) or not-fun (unsafe)–and now maintained by the BGC through their adoption of the campaign–risks undermining what is a far more complex picture that requires a more nuanced approach similar to that taken to address other harmful, addictive products, and the industries that produce and market them [26]. Yet they also referred to slipping down paths, or transitioning, to addiction, which represents an inconsistency in their framing. We also found little evidence to support the claims about the campaign’s effectiveness and the results of our critical appraisal questions whether their surveys can be considered a robust evaluation from which conclusions about effectiveness can really be drawn, rather than a form of market research. Even though the ASA ruled against them, the Senet Group continued to claim a positive impact of the campaign in multiple fora [44, 76].

The Senet Group’s framing of the problem, its causes, and proposed remedies, which underpin the *When the Fun Stops*, *Stop* campaign, do not reflect public health approaches but appear to align closely with what has been found in previous research on industry-funded initiatives, including those falling within the corporate social responsibility (CSR) concept [19, 21, 28, 52, 77–79]. These include: (1) portraying the problem as confined to a minority, minimising the scale of the problem, and asserting that most consumption is enjoyed safely and in moderation [52]; (2) framing the issue in the context of personal control, individualised levels of safe use, and the responsibility of individuals to consume for enjoyment and in moderation [77, 80], and (3) promotion of education and awareness campaigns to support responsible use and self-control by the majority, while specific interventions are confined to the minority who are harmed by their consumption [52, 81]. In her critical ethnography of machine gambling in Las Vegas, Schüll notes the contradiction between industry claims that most people are not at risk of addiction while simultaneously providing responsible gambling messaging that suggest all consumers adopt the risk management techniques outlined in these messages with the aim of controlling risks that by implication everyone is exposed to when gambling [8].

The Senet Group also asserted the need for industry-led interventions, such as public awareness campaigns, even though these have often been found to promote the product or industry in question [27–30], a rejection of government regulation as ineffective compared to self-regulation and voluntary agreements, and the manipulation of the concepts of culture, freedom, choice and consumer demand, as when, for example, consumers are encouraged to ‘make friends’ with gambling. The promotion of self-regulation and employment of concepts of freedom and choice are documented among other industries and CSR bodies [82, 83]. Furthermore, while there are few academic studies of the gambling industry, two previous studies have demonstrated similarities between the strategies adopted by other harmful industries, such as tobacco and alcohol, and those employed by the gambling industry. Hancock et al analysed the corporate political activity of the Australian gambling industry revealing the use of tactics, strategies and arguments previously identified by research on the tobacco and alcohol industries [84]. Petticrew et al demonstrated cross-industry, including the gambling industry, manipulation of the concept of complexity to influence the conceptualisation of public health issues and to argue against the adoption of evidence-based population-level policy interventions [85].

The Senet Group portrayed their campaign as underpinned by research, referring to an evaluation conducted between 2015 and 2017. They claimed that their ideas and campaign were informed by evidence and knowledge of what constitutes effective public health messaging, endorsing the use of humour and asserting that government-led interventions are inferior to using peer-pressure. This is not supported by the evidence on the effectiveness of such approaches. For example, mandated tobacco health warnings contain graphic imagery that leverages disgust and fear [86, 87], enhanced by revolving images (as opposed to the Senet Group’s and now the BGC’s continuing use of the same material) and plain packaging (as opposed to appearing alongside company marketing) [88, 89]. Pictorial warnings have been shown to be more effective than text-only warnings in relation to both impact and longevity [90]. The effect of humour in health messaging is complex and variable depending on the make-up of the audience (including age and gender), the channel and formats used for dissemination, as well as the message content [91–94]. It is known that, without careful consideration of the evidence on messaging and framing impacts, messages can “backfire”, rendering campaigns ineffective or even detrimental [95]. Indeed, as explained in the introduction, evidence of backfire has been demonstrated previously, whereby study participants who were exposed to the yellow-coloured version of the campaign’s message were found to bet a greater proportion of their money compared to those individuals not exposed to the messaging [46]. The content of responsible gambling messaging is known to be ambiguous and potentially less effective than messages about the risks of gambling or those that aim to correct erroneous beliefs [96]. Interventions that rely on individuals to use their personal resources, or *agency*, to address significant public health issues are also least likely to be effective, and more likely to deepen inequities [97]. Such approaches continue to be promoted and adopted despite their failure to reduce health inequities, as they maintain the status quo, are easy to ‘sell’ to the public, serve powerful vested interests and minimise legal liability to producers [98].

By framing the issue as affecting a minority who are weak or prone to addiction compared to a majority comprised of “millions” who enjoy gambling safely, the Senet Group’s framing potentially contributed to the perception of the problem as one of a separate minority group in need of professional help–as opposed to a wider public health issue. This has important implications for public health. A substantial body of evidence exists to show that when individuals are provided with information that creates, even if false, the perception that an activity or product is very common, thereby creating ‘social norms’, then they tend to feel unusual if they are not engaging with it, which in turn influences their behaviour [99]. Such framing also potentially deflects attention away from the likelihood that many regular consumers of electronic gambling machines will experience harm from their use of such high-speed products [7]. It also overlooks the evidence on the limitations of current population surveys to accurately enumerate those experiencing problem gambling [100]. The assertion that only a small number of people who gamble experience harm has been described as “at best, simplistic and misleading” [7].

### Strengths and limitations

To our knowledge, this is the first analysis of the provenance, ideational and evidence base of a major industry-funded gambling awareness campaign. However, there may be additional unpublished data that we could not locate. We also cannot comment on the motivation for adopting certain framings or how effective they have been. Future research could explore the influence industry-supported groups have on policy and public opinion, for example, and how particular framings are adopted by the mass media and in policy discourse. To our knowledge, no independent research assessing the unintended impacts of the campaign has been conducted. This is particularly relevant for children, who may be exposed to the campaign through land-based gambling venues, and mass and social media, for example when using apps [101]. Further research should attempt to establish, whether or not, through its use of colour, imagery, visual effects and music, the word ‘fun’, as well as the alignment with culture (e.g. football) and tradition (e.g. multiple generations and a stereotypical British household), the campaign potentially markets or endorses gambling as has been suggested by different commentators, and captured, for example, by one author of an article in the Yorkshire Post:

“"When the fun stops, stop." Really?…Can you imagine the authorities allowing Big Tobacco to present smoking in the same "fun" way, knowing everything we do today about the dangers of inhaling the fumes from burning tobacco? When the cancer starts, stop?” [102].

### Implications

Here we have demonstrated that the dominant framing adopted by the Senet Group aligned with industry interests and resembled those employed, for several decades, by other industries which also sell harmful products and those they support as a form of CSR. It reflects wider challenges in the UK and internationally, that is, the mismatch between interventions which the evidence suggests will be effective in preventing gambling harm, and those that are delivered in practice, and the role of industry and industry-funded bodies in the design and delivery of health information and interventions [103].

Although the Senet Group, and others, acknowledged the need for new messaging campaigns that go beyond *When the fun stops*, *stop*, the Senet Group continued to assert the success of the campaign and announced that it intended to launch a “next generation” of the campaign [63]. While dissemination of the campaign continues and the BGC has asserted that “the legacy they [The Senet Group] leave through the Safer Gambling Commitments, which provide a roadmap for raising standards across our industry” [41], our findings support that the ongoing use of the campaign and framing of the Senet Group as a previous industry standards ‘watchdog’ from which to build upon need to be questioned. The Senet Group’s framing aligned with industry interests, they were fully funded by the gambling industry thereby inducing financial conflicts of interest to their activities, and there is no evidence that the campaign has been effective from a public health perspective. Overall, our findings, in combination with other research that calls into question the effectiveness of current forms of responsible gambling promotion [96, 103], build the case for careful review of their use and impacts in all regions.

## Conclusions

The Senet Group’s framing of the problem, its causes, and responses deemed acceptable resemble those adopted by other industries who produce and sell harmful products, and the groups they fund, when seeking to influence policy and issue-framing [85]. Our analysis extends these findings to a UK-based gambling industry-funded body. The findings also bring into question claims made by the Senet Group in relation to reducing gambling harms and serving as a “Gambling watchdog” [72].

Our findings have implications for gambling-related policy and public health practice, while contributing to a growing body of evidence on the impacts and implications of industry involvement in addressing the issues caused by their products. To minimise conflicts of interest and barriers to progress, public health interventions that seek to address gambling-related harms, such as public education campaigns, should be designed, implemented, and evaluated independent of industry and industry-funded organisations.

## Supporting information

S1 File Appraisal of Senet Group-commissioned campaign evaluation. (Performed using open-access tool: Downes MJ, Brennan ML, Williams HC, Dean RS. Development of a critical appraisal tool to assess the quality of cross-sectional studies (AXIS). BMJ Open. 2016;6(12):e011458) [57].
(PDF)
