# Supplementary material for: “When the Fun Stops, Stop”: An analysis of the provenance, framing and evidence of a ‘responsible gambling’ campaign
Source: PLoS One. 2021 Aug 26;16(8):e0255145. doi: 10.1371/journal.pone.0255145 (PMC8389453; doi:10.1371/journal.pone.0255145)
Supplement: S1 File — (Performed using open-access tool: Downes MJ, Brennan ML, Williams HC, Dean RS. Development of a critical appraisal tool to assess the quality of cross-sectional studies (AXIS). BMJ Open. 2016;6(12):e011458) [57]. (PDF) [file pone.0255145.s001.pdf]

Appraisal of Senet Group-commissioned campaign evaluation

(performed using open-access tool: Downes MJ, Brennan ML, Williams HC, Dean RS. Development of a critical appraisal tool to assess the quality of cross-sectional studies (AXIS). *BMJ Open*. 2016;6(12):e011458)

Where different answers were provided by independent reviewers, both answers are presented in the table.

	Question	Yes	No	Don't know/ Comment
Introduction				
1	Were the aims/objectives of the study clear?		**No**	Beyond stating that the study aimed to evaluate the campaign there were no clear aims or objectives listed. The phrasing of the findings sections suggests that the aim was to identify positive messages about the campaign.
Methods				
2	Was the study design appropriate for the stated aim(s)?			**Do not know:** as multiple aims for the campaign were stated by the Senet Group the appropriateness of the study design is unclear. However, the study did not objectively measure behaviour change over time, nor is it appropriate to support extrapolating to the entire adult population.<br><br>**No.** It is based on a simple survey. One of the weakest study designs.
3	Was the sample size justified?		**No**	It is stated that a representative sample was obtained.<br><br>No justification for the sample size was given and sub-population analyses were performed without stating whether these sub-groups remained representative.
4	Was the target/reference population clearly defined? (Is it clear who the research was about?)		**No**	The analysis demonstrates that different target audiences have been stated by the Senet Group.
5	Was the sample frame taken from an appropriate population base so that it closely represented the target/reference population under investigation?			**Do not know:** the ambiguity of the aims of the campaign and the target audience hinder critique of who should be sampled or over-represented in the sample frame. Of note, the sample does not allow for extrapolation to the entire adult population and may also not be representative when the sub-group analyses were performed.

6	Was the selection process likely to select subjects/participants that were representative of the target/reference population under investigation?			**Do not know:** no refence was made to potential selection biases<br><br>**No**
7	Were measures undertaken to address and categorise non-responders?		**No**	Nothing was stated about this in the evaluation reports.
8	Were the risk factor and outcome variables measured appropriate to the aims of the study?			**Do not know:** the ambiguity of the aims of the campaign and the target audience hinder critique of this aspect of the study. It is also unclear if important risk factors and outcome variables, such as socio-economic, employment and health status were measured.<br><br>**No**
9	Were the risk factor and outcome variables measured correctly using instruments/measurements that had been trialled, piloted or published previously?			**Do not know:** see above answer to Q8
10	Is it clear what was used to determined statistical significance and/or precision estimates? (e.g. p-values, confidence intervals)		**No**	No analysis is presented.
11	Were the methods (including statistical methods) sufficiently described to enable them to be repeated?		**No**	No methods are presented.
Results				
12	Were the basic data adequately described?		**No**	Only sample sizes were provided
13	Does the response rate raise concerns about non-response bias?			**Do not know:** no responses rates were reported
14	If appropriate, was information about non-responders described?		**No**	Information about non-responders was not described
15	Were the results internally consistent?		**No**	Sub-populations of the entire sample are used for different analyses without explanation
16	Were the results presented for all the analyses described in the methods?			**Do not know:** No analyses are presented, and the evaluation report for the latter half of 2015 could not be located.

Discussion				
17	Were the authors' discussions and conclusions justified by the results?		**No**	No evidence could be found to substantiate the claims made by the Senet Group about the effectiveness of the campaign in terms of behaviour change and at the level of the general adult population, or in comparison to other comparable public health campaigns. Over-interpretation of findings.
18	Were the limitations of the study discussed?		**No**	
Other				
19	Were there any funding sources or conflicts of interest that may affect the authors' interpretation of the results?	Yes		The Senet Group is funded by the gambling industry. The evaluation was commissioned by the Senet Group to evaluate their campaign. It is unclear what influence the Senet Group had on the design of the evaluation or on the reporting of the results. It was conducted by a market research company which does work for gambling companies.<br>Note that all results are accessed through the Senet Group website suggestive of editorial control of what is publicly released.
20	Was ethical approval or consent of participants attained?			**Do not know:** No mention of attaining ethical approval or consent was identified.
